# Unusual malignant cause of adult intussusception: Stromal tumor of the small bowel

**DOI:** 10.4103/0974-2700.66544

**Published:** 2010

**Authors:** Khalid Rabbani, Youssef Narjis, Benacer Finech, Abdelhamid Elidrissi

**Affiliations:** Department of General Surgery, University Caddi ayyad, Marrakech, Morroco

Sir,

Gastrointestinal stromal tumors (GISTs) are rare and specific tumors of the gastrointestinal system. They are commonly located in the stomach and small bowel. Intussusception and obstruction is a very uncommon presentation of these lesions because of their tendency to grow in an extraluminal fashion.

**Figure 1:**
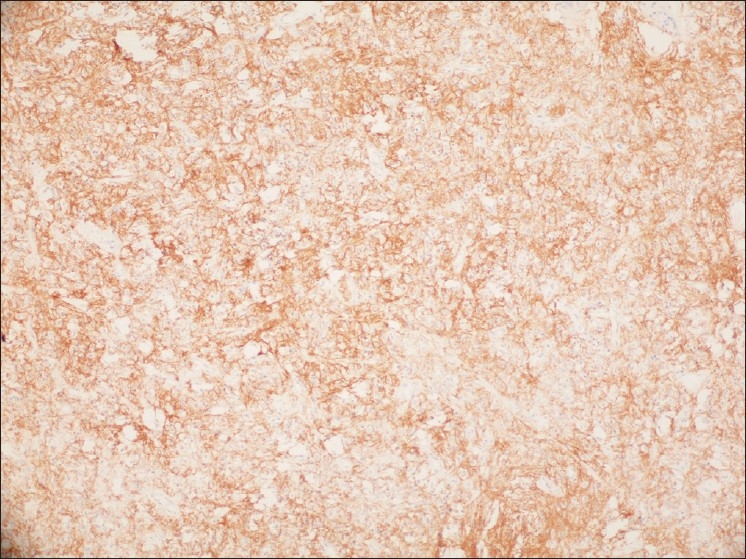
Set marker “c-KIT” showing the cytoplasm of reactive cells stained brown

A 59-year-old female patient was admitted to the emergency room with a 6-month history of intermittent attacks of abdominal distension and pain, obstipation, and new onset of vomiting. Physical examination revealed abdominal distension and hyperactive bowel sounds. No significant weight loss or palpable mass was identified. Computed tomographic scan showed the “target” sign of intussusception. Laparotomy revealed ileoileal intussusception secondary to intramural mass in the terminal ileum, located 70 cm proximal to the ileocecal valve. Ileoileal anastomosis was performed after resection of the tumoral segment. No complication occurred in the early post-operative period. Pathological investigation confirmed that the neoplasm was a small bowel GIST. Immunohistochemical studies showed positive stains for protein S100, vimentin and c-kit and CD 34 [Figure 1].

GISTs are a group of rare tumors of the digestive tract that constitute about 1% of all gastrointestinal cancers;[1] in 20–30% of the patients, it has been seen in the small intestines. Notwithstanding this, GISTs are the least common of small intestinal malignant neoplasms and, because of their insidious presentation, they are often not suspected prior to surgery. Consequently, their diagnosis is often delayed or even overlooked, and usually it is made after laparotomy and formal pathologic examination.[2] Small bowel tumors are uncommon and can have a long delay prior to diagnosis because they are usually asymptomatic, especially in their early stages, and they often go unrecognized until severe symptoms ensue, which can create surgical emergencies.[2,3] GISTs tend to grow in an extraluminal fashion; however, they can also erode into the lumen of the gastrointestinal tract, inducing significant hemorrhage or anemia from occult bleeding.[4] They can also rupture into the peritoneal cavity causing significant hemorrhage.[5] In addition to symptoms from mass effect or bleeding, GISTs can cause intussusception or, rarely, intestinal obstruction.[1,5] In this case, the patient was asymptomatic until intestinal obstruction developed. Radiological studies such as computed tomographic scans, barium studies, abdominal US, plain film and radionuclide studies may be useful in its pre-operative diagnosis.[1,3] Intussusception is correctly diagnosed pre-operatively in only one-third of the cases. Enteric intussusception is characterized in longitudinal section by an oval “tumor,” which, in cross-section, has a “target” appearance with infolding of bright luminal interfaces giving multiple concentric rings, which was present in this case. Their biological behavior is difficult to predict, ranging from benign to malignant, thus rendering their size and mitotic index the most reliable prognostic factors.

Several mutations in the c-kit gene have been incriminated in the oncogenesis of GIST,[5,6] leading to constitutive expression of the receptor tyrosine kinase involved in cell differentiation and proliferation. Expression of this tyrosine kinase c-kit (CD117), demonstrated by immunohistochemistry, has been shown to be a sensitive and specific diagnostic marker for GIST.

Surgical resection is recommended in nearly all cases of adult intussusception inducted by GISTs because of the high prevalence of structural anomalies and the relatively high risk of the underlying malignancy.[6]

This case presents an unusual malignant cause of adult intussusception tumors of the small intestine that may have a malignant potential. Radical surgical resection is essential and will determine the overall outcome.
